# Supplementary material for: Poly(Ethylene Furanoate) along Its Life-Cycle from a Polycondensation Approach to High-Performance Yarn and Its Recyclate
Source: Materials (Basel). 2021 Feb 23;14(4):1044. doi: 10.3390/ma14041044 (PMC7926444; doi:10.3390/ma14041044)
Supplement: Supplementary file 1 [file materials-14-01044-s001.pdf]

materials

Supplementary Material to

Poly(Ethylene Furanoate) along its Life-Cycle from a Polycondensation Approach to High-Performance Yarn and its Recyclate

Tim Höhnemann, Mark Steinmann*, Stefan Schindler, Martin Hoss, Simon König, Antje Ota, Martin Dauner*, and Michael R. Buchmeiser

S1: Nomenclature of samples

The samples used in the manuscript are defined as follows:

- PEF granules are consecutively numbered: PEF-01 to PEF-09
- LOYs and POYs have the number of their respective PEF-granule: *e.g. POY from PEF-03: "POY-03"*
- Two POYs of the same PEF get the annotation a and b, *e.g. "LOY-03a" and "LOY-03b", both based on PEF-03*
- FDYs have the number of their respective PEF-granule as well + the annotation "L" or "P" indicating if the respective yarn is drawn from LOY or POY., *e.g.: FDY from POY-03: "FDY-P-03".*

Shear rheological characterization of PEF-03, -04 and 05.

A reduced strength was obtained for FDY-3, which was spun at 260 °C from POY-2, while FDY-4, spun at 275 °C had a slightly higher tenacity than FDY-2, spun at 260 °C. This effect stands against the trend of *[η]* within the three lots (0.747 → 0.780 → 0.790 dL g^{-1}), but can be correlated with their shear-rheological characterization, shown in Figure S1.

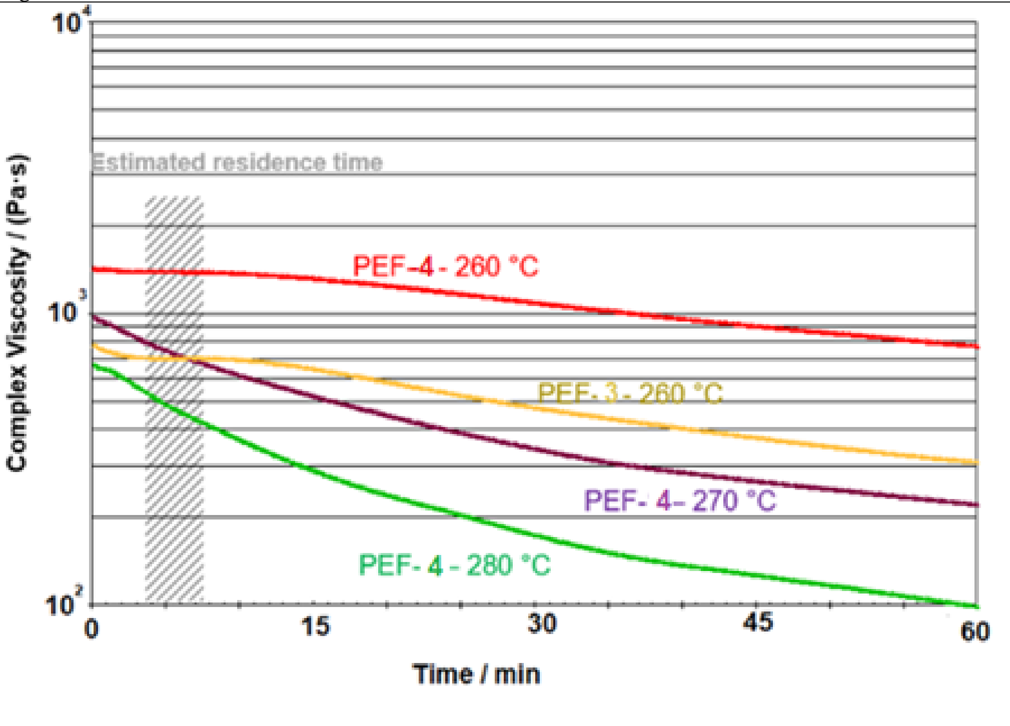

(a)

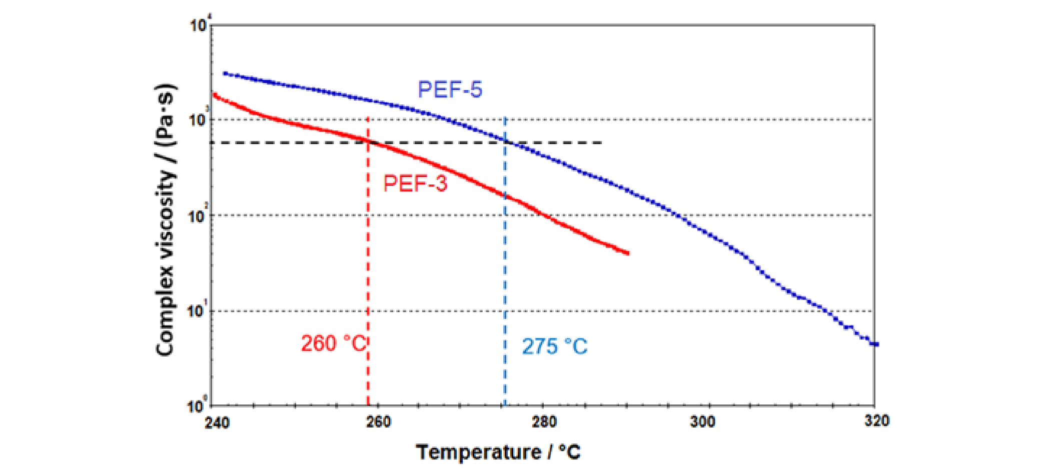

(b)

Figure S1. Shear rheological characterization of different PEF-lots after SSP. (**a**) Time-sweep of PEF-03 and -04 at different temperatures. (**b**) Temperature sweeps of PEF-04 and -06.

Compared to the small changes in *[η]* (Table 1), the shift of the complex viscosity was more pronounced. Opposing the time sweeps of PEF-3 at the used spinning temperature of 260 °C and the curves of PEF-4 at 260 °C, 270 °C and 280 °C Figure S2a and the temperature sweeps for PEF-3 and PEF-05 in Figure S2b, the optimal process window shifted to higher temperature (PEF-03: 260 °C → PEF-04: 270 °C → PEF-05: → 275 °C). Thus, a higher spinning temperature would have been more suitable for PEF-04 to gain the best possible filament mechanics, such as achieved with PEF-05.

Processing steps of PEF

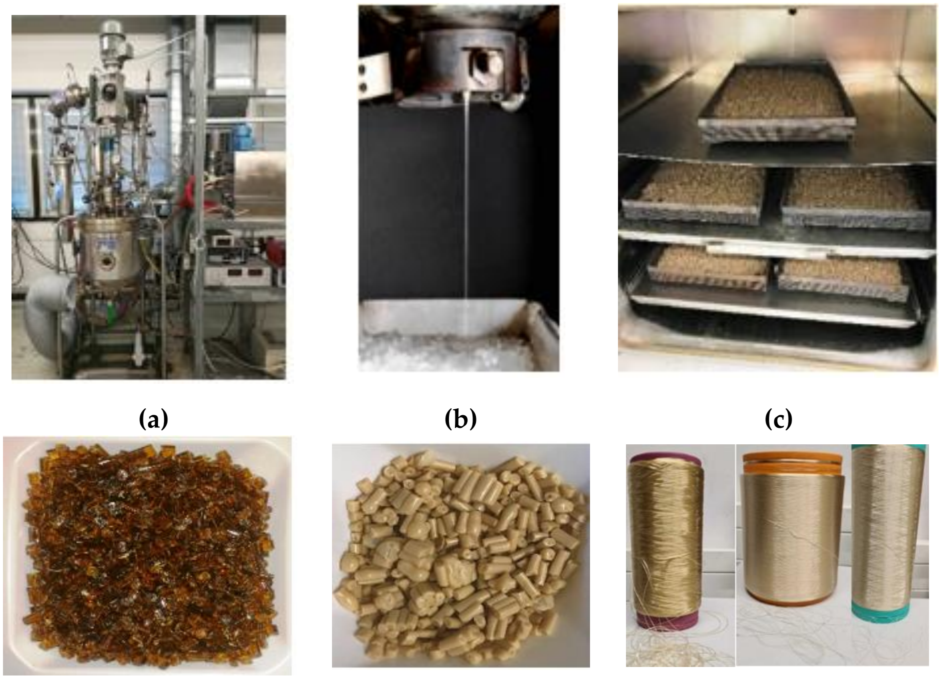

(a) **(b)** **(c)**

(d) **(e)** **(f)**

Figure S2. Illustration of the process steps of PEF from synthesis to FDY (**a**) Reactor; (**b**) Release of PEF melt from reactor; (**c**) SSP oven; (**d**) pre-SSP granule; (**e**) post-SSP granule; (**f**) PEF yarn: LOY/POY/FDY (left to right).

DSC-curves

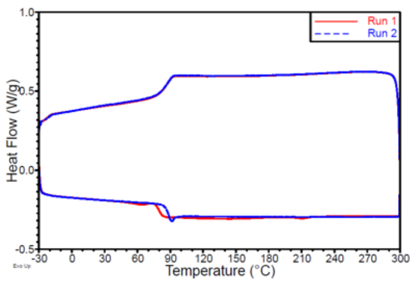

(a)

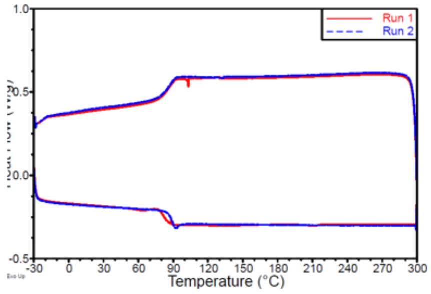

(b)

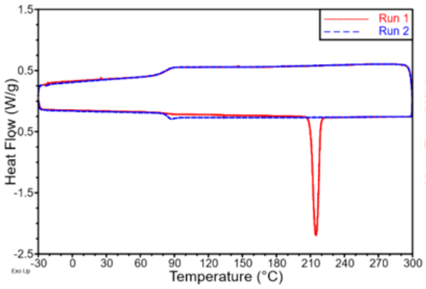

(c)

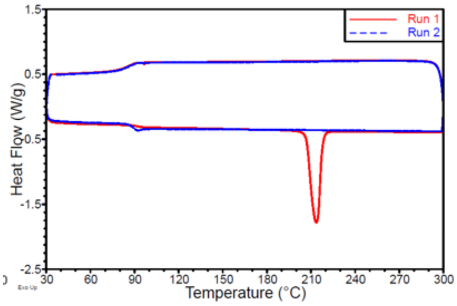

(d)

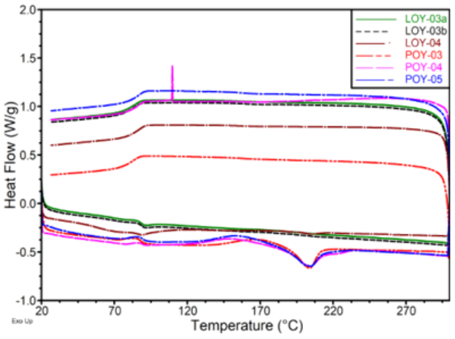

(e)

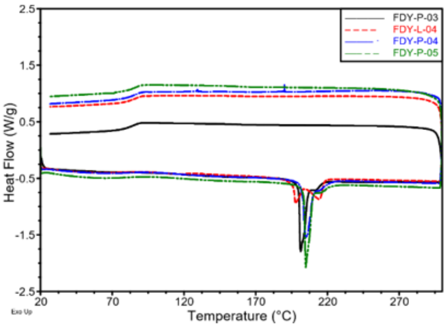

(f)

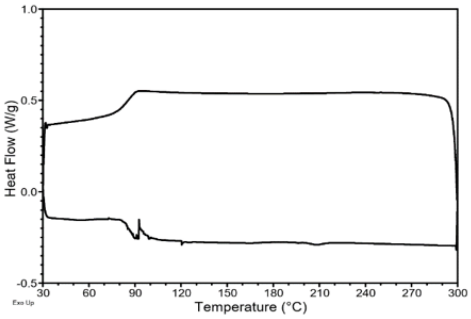

(g)

Figure S3. DSC curves of PEF granules and yarns at different process steps. (**a**) PEF-03 (pre-SSP) (**b**) PEF-04 (pre-SSP) (**c**) PEF-03 (post SSP) (**d**) PEF-04 (post SSP) (**e**) PEF LOYs/POYs (**f**) PEF-FDYs (**g**) PEF-03-recyclate.

Tensile test data

FDY-P-03	Max. Force	Strain	Tensile strength	Work uptake	Modulus 0.5 %	Modulus 0 - 1%
	cN	%	cN/tex	cN·cm	N/tex	N/tex
Mean:	397,9	4,7	63,0	525,2	15,14	16,27
stdev:	18,8	0,2	3,0	53,4	0,64	0,78
Min:	365,5	4,1	56,4	399,6	14,13	14,64
Max:	427,4	5,1	67,6	615,7	16,18	17,74
CV:	4,83%	4,84%	4,71%	10,16%	4,22%	4,82%

(a)

FDY-L-04	Max. Force	Strain	Tensile strength	Work uptake	Modulus 0.5 %	Modulus 0 - 1%
	cN	%	cN/tex	cN·cm	N/tex	N/tex
Mean:	1593,0	7,4	34,7	4328,0	10,28	9,87
stdev:	51,8	2,9	1,1	2216,0	0,34	0,19
Min:	1507,0	4,4	32,8	2078,0	9,46	9,49
Max:	1677,0	14,0	36,5	9557,0	10,77	10,24
CV:	3,25%	39,22%	3,25%	51,21%	3,31%	1,96%

(b)

FDY-P-04	Max. Force	Strain	Tensile strength	Work uptake	Modulus 0.5 %	Modulus 0 - 1%
	cN	%	cN/tex	cN·cm	N/tex	N/tex
Mean:	434,9	5,1	53,0	606,4	12,74	11,95
stdev:	14,5	0,3	1,8	52,5	0,60	0,51
Min:	409,5	4,8	49,9	544,6	11,31	10,64
Max:	463,2	5,6	56,5	710,6	13,85	12,79
CV:	3,32%	4,91%	3,32%	8,66%	4,67%	4,28%

(c)

FDY-P-05	Max. Force	Strain	Tensile strength	Work uptake	Modulus 0.5 %	Modulus 0 - 1%
	cN	%	cN/tex	cN·cm	N/tex	N/tex
Mean:	514,1	6,0	62,0	861,4	13,66	13,01
stdev:	6,3	0,1	0,8	33,5	0,35	0,18
Min:	489,9	5,8	60,2	816,5	13,09	12,66
Max:	525,1	6,3	63,3	928,3	14,45	13,23
CV:	1,23%	2,16%	1,23%	3,89%	2,54%	1,35%

(d)

Figure S4.1: Tensile test data (summary). (**a**) FDY-L-04 (**b**) FDY-P-03 (**c**) FDY-P-04 (**d**) FDY-P-05.

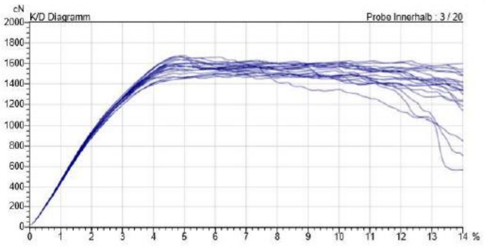

(**a**)

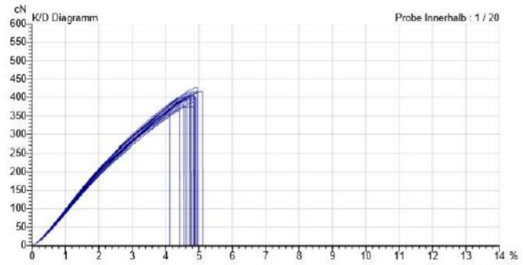

(**b**)

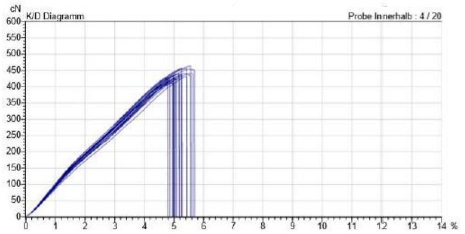

(**c**)

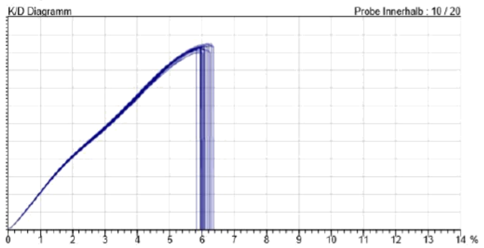

(**d**)

Figure S4.2: Force-strain curves of PEF-FDY. (a) FDY-L-04 (b) FDY-P-03 (c) FDY-P-04 (d) FDY-P-05.

Annotation: Stress values in the tables can slightly derive from the reported values in the manuscript, because multiple yarns were tested in a row with the same titer input. The stress was afterward corrected to the respective titer of the sample.

Peak deconvolution method (XRD)

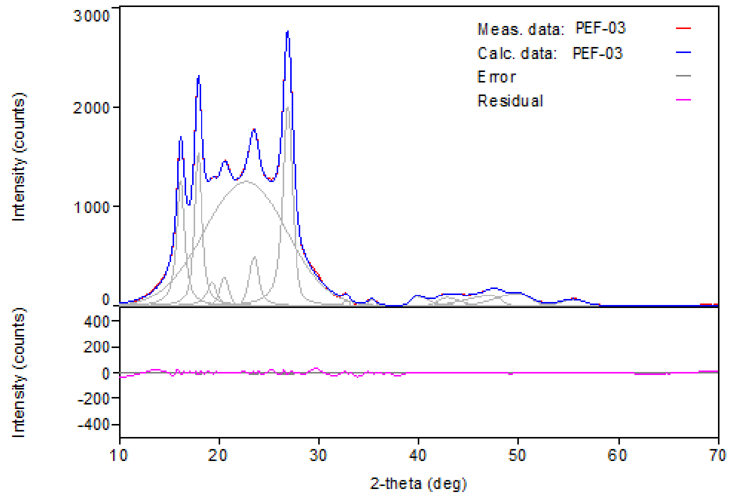

(a)

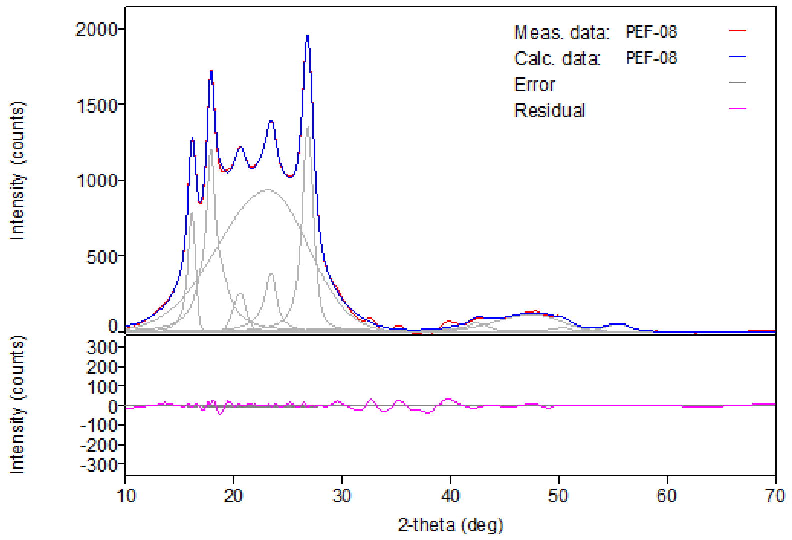

(b)

Figure S5 Peak profile analysis (**a**) PEF-03 (**b**) PEF-08.

Degree of preferred orientation of FDY.

In Figure S7 the azimutal scan of the [020] lattice plane for all FDY derived fibers is shown. Based on that, the calculations of the Hermanns orientation factor was performed in the following.

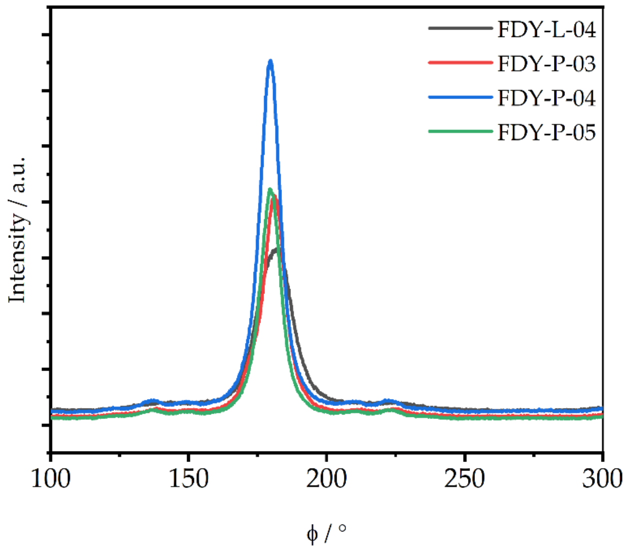

Figure S6.1: Azimuthal intensity scan of the FDY samples.

The calculation of the Hermanns orientation factor f_c [65] was calculated based on azimutal scan of [002] lattice plane by using the following equation (eq. SI).

$$f_c = \frac{3\ \langle cos^2\phi\rangle - 1}{2}, \text{ whereas } \langle cos^2\phi\rangle = \frac{\int_0^{\pi/2} I\ (\phi)\cdot cos^2\phi\ \cdot \sin\phi\ d\phi}{\int_0^{\pi/2} I(\phi)\cdot sin\phi\ d\phi} \tag{S1}$$

The resulting values are given in the Table S1.

Table S1: Hermanns orientation factors f_c of the FDYs.

Sample:	FDY-L-04	FDY-P-03	FDY-P-04	FDY-P-05
f_c:	0.90	0.93	0.94	0.87

As the experimental error can be approximated to lie around 5%, the differences of the samples lie within and no conclusions to the differences in the mechanics can be drawn.

Structural and microstructural analysis by XRD

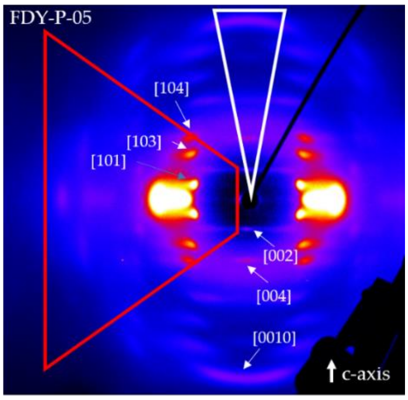

Figure S7.1: Diffraction image of FDY-P-05 fiber with identified crystal planes ([hkl]).

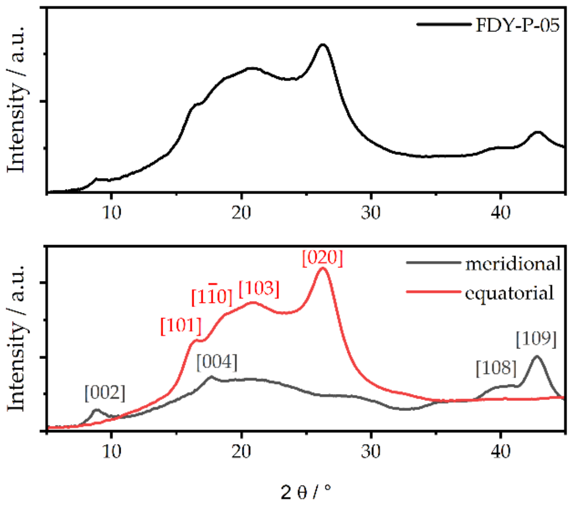

(a)

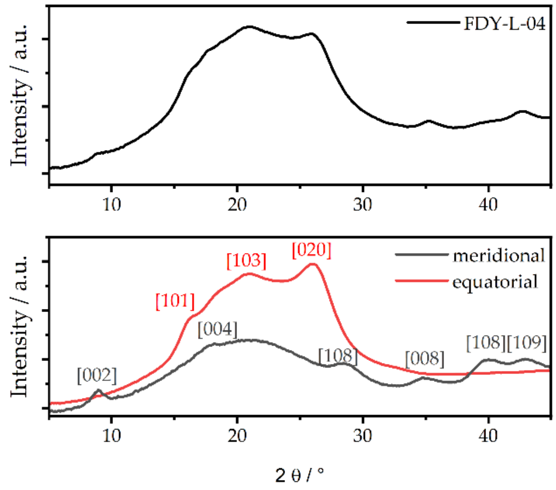

(b)

Figure S7. 2: X-ray pattern of the meridional and equatorial lattice planes ([hkl]) for (**a**) POY- and (**b**) LOY-derived FDY yarns.

References

1. [65] White, J.L., Spruiell, J.E. The specification of orientation and its development in polymer processing. *Polym. Eng. Sci.* 1983, 23, 47–256.
